# Mechanical Circulatory Support in Management of Cardiogenic Shock and Myxedema Coma

**DOI:** 10.1155/2019/2595736

**Published:** 2019-03-06

**Authors:** Jelena Z. Arnautovic, Randi Connor-Schuler, Randy Ip

**Affiliations:** ^1^Division of Cardiovascular Medicine, St. John Providence Ascension Health System, Warren, MI, USA; ^2^Department of Emergency Medicine, Henry Ford Health System, Detroit, MI, USA

## Abstract

The cardiovascular system is a major target of thyroid hormone action and the two systems are closely interlinked. It can be greatly impacted even with subtle alterations in thyroid function. Caution is needed when implementing thyroid hormone replacement in patients with severe hypothyroidism, especially in the setting of ischemic coronary artery disease. If not properly treated, myxedema may ensue. Given the high mortality of myxedema coma, supportive care may not always suffice and patients may require more invasive interventions. We present a challenging case of a patient with overt hypothyroidism with concurrent acute coronary syndrome which subsequently lead to myxedema coma and cardiogenic shock. A transcaval approach for the delivery of an Impella 5.0 (Abiomed Inc., Danvers, MA) was utilized in supporting this patient. To our knowledge, this is the first reported case that describes the use of a mechanical circulatory support in treating myxedema-induced cardiovascular collapse.

## 1. Introduction

Thyroid function and the cardiovascular (CV) system have an intricate relationship as patients with untreated thyroid dysfunction may develop an accelerated onset of symptomatic CV disease [1]. Modifiable atherosclerotic risk factors, including hypercholesterolemia, diastolic hypertension, increased carotid intimal-media thickness, and reduced endothelial nitric oxide, accompany overt hypothyroidism and could be reversible with proper TH replacement [2]. Although there is a close relationship between thyroid function and cardiovascular health, there have been no interventional studies of TH replacement in patients with acute myocardial infarction that have been published to date, therefore making a causal relationship between thyroid dysfunction and outcomes difficult to ascertain [1].

We present a challenging case of a patient with overt hypothyroidism complicated by myxedema with concomitant ischemic heart disease. This case is also remarkable for the use of a transcaval access for the delivery of an Impella 5.0 (Abiomed Inc., Danvers, MA) in supporting myxedema-induced cardiovascular collapse, which to our knowledge has not been previously documented.

## 2. Case Report

A 41-year-old male, without regular medical care, initially presented in the outpatient setting with progressive fatigue, weight gain, shortness of breath, and lower extremity edema over the past year. At that time, he was diagnosed with hypothyroidism (TSH 136 uIU/mL) and was started on 50 *μ*cg PO levothyroxine daily. Two days later, he presented to the emergency room with chest pain and worsening shortness of breath. The patient was admitted for further evaluation which included an ischemic workup for coronary artery disease.

A diagnostic cardiac catheterization was performed and the patient was noted to have multivessel obstructive coronary disease with a severely reduced ejection fraction. The patient subsequently underwent stenting of the left anterior descending and left circumflex coronary arteries. However, within 24 hours, the patient developed cardiogenic shock and a second percutaneous intervention was emergently done to address the right coronary artery lesion. Due to patient's condition, an intra-aortic balloon pump (IABP) was utilized and he was transferred to another institution for escalation of care.

Upon arrival to the second institution, vital signs demonstrated a blood pressure of 67/31 mmHg, a heart rate of 68 bpm, an oral temperature of 35.7°C, a respiratory rate of 14, and an oxygen saturation of 99% on 4 L nasal cannula. Evaluation of the patient was significant for altered mental status and signs of systemic hypoperfusion with cold extremities in the lower extremities. The physical exam also was positive for bilateral nonpitting edema in all extremities. Further pertinent positives on the physical exam were notable for thinned hair to the lateral eyebrows, macroglossia, a waxy, yellow appearance to his skin, and an absence of hair on the lower extremities.

**Figure 1:**
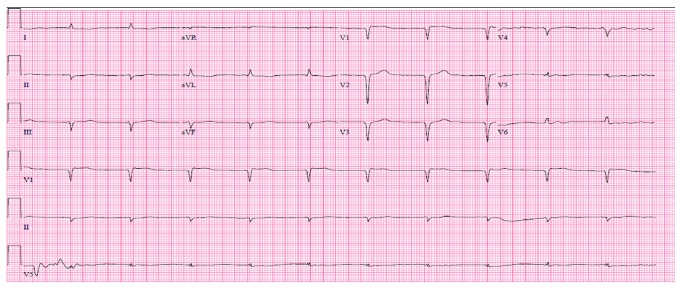
Admission electrocardiogram. Low-voltage junctional bradycardia with anteroseptal and inferior Q waves.

Initial laboratory findings included hemoglobin of 7.7 g/dL, platelets of 24 K/*μ*L, and a TSH level of 51.09 uIU/mL with free T4 of 0.26 ng/dL and free T3<1.0 pg/mL. Thyroid peroxidase was also noted to be elevated at 209 IU/mL (normal < 9 IU/mL). An electrocardiogram was obtained which showed diffuse Q waves (Figure 1). Cardiac monitoring was reviewed which demonstrated low-voltage complexes with an intermittent junctional bradycardia. Bedside echocardiogram revealed severely reduced systolic ejection fraction of 10% with mild to moderate RV dysfunction.

Patient's clinical presentation was consistent with myxedema coma, and the patient was treated with stress-dose steroids and intravenous levothyroxine. Given the refractory cardiogenic shock, the IABP was upgraded to a transcaval Impella 5.0 upon admission. Patient's clinical condition subsequently improved as his lactate cleared from 5.5 mmol/L to 1.1 mmol/L; vasopressors were discontinued; Impella wean commenced over the course of a few days. Unfortunately, his clinical course was complicated with acute ischemia of his right lower extremity on day 6 leading to acute renal failure and sepsis. Ischemia was likely multifactorial with a large 24F venous sheath from the Impella exerting pressure on the femoral artery also containing 5F arterial line; the patient also developed an aortic thrombus further impairing perfusion. Despite emergent revascularization efforts and Impella removal, the muscles were nonviable. Family was informed of the need for an above the knee amputation; however, the family decision was to proceed with comfort care and the patient died on the 8th day of hospitalization.

## 3. Discussion

Thyroid function plays a significant role in the health of the cardiovascular system. Thyroid hormone activates cytoprotective mechanisms, stimulates cell growth, and promotes neoangiogenesis and metabolic adaptation. In the acute myocardial infarction setting, optimal thyroid function aids in cardioprotection by reducing myocardial damage and reversing left ventricular (LV) remodeling [3]. A recent meta-analysis showed that patients with subclinical hypothyroidism are at higher risk of developing cardiovascular events, particularly with TSH levels > 10 *μ*U/L, and patients with untreated hypothyroidism are at an increased risk of bradycardia, systolic and diastolic dysfunction, and increased peripheral vasoconstriction [3, 4].

Patients with newly diagnosed severe hypothyroidism and significant cardiovascular risk factors may present a difficult challenge for the clinician. Great caution is needed when implementing TH replacement in this subset of patients, as thyroxine (T4) therapy increases the metabolic demands of the body which can provoke myocardial ischemia. Patients at risk for ischemic heart disease may require risk stratification for coronary disease. If coronary insufficiency is apparent, prompt management should be implemented to improve the oxygen supply to the ischemic myocardium before increasing the myocardial demand with T4 [5].

It was likely that our patient infarcted prior to initial presentation to the outside facility and had a preexisting cardiomyopathy from long-standing, untreated, hypothyroidism in addition to underlying ischemic disease. Patient's presentation may be explained by cardiogenic shock alone. However, patient's preexisting cardiac insufficiency may have been further exacerbated after the initiation of T4 therapy along with an additional iodine load from the cardiac catheterization. This may have triggered a cascade of events that lead to the development of myxedema coma complicating the treatment plan.

The incidence of myxedema is roughly 0.22 per million people per year with mortality rates approaching 30-60% with treatment [6]. It can develop idiopathically or after additional stresses to the body such as infection, stroke, iodine contrast, and myocardial infarction in patients with preexisting hypothyroidism, as presented in our study [7, 8]. A common source of excess iodine exposure in many patients results from the use of iodinated contrast media (ICM) for radiological studies and procedures such as coronary angiography, which is well tolerated in most euthyroid individuals but may lead to thyroid dysfunction in susceptible groups [9, 10]. The prevalence of thyroid dysfunction after coronary angiogram contrast exposure is not very well described, and no specific guidelines exist [9]. Several studies have shown a clear and definitive association between ICM exposure and the development of overt hypothyroidism [9, 11, 12]. Case control studies suggest that ICM exposure at least doubles the risk of subsequent overt hyperthyroidism and triples the risk of overt hypothyroidism [9], and Lee et al. noted that 22% of their study population developed an abnormal TSH within one to four weeks of receiving an elective computed tomography scan [13].

The main mechanism for iodine contrast-induced hypothyroidism is known as a failure to escape the Wolff-Chaikoff effect, first described in 1948 [14]. This effect is usually transitory and does not typically lead to overt hypothyroidism. This escape process is mainly due to the downregulation and reduction in sodium/iodide symporter (NIS) expression resulting in decreased iodine transport into the thyroid and subsequent resumption of thyroid hormone synthesis, typically occurring 24 hours after excess iodine exposure [15–17].

Monitoring of thyroid function should be considered especially in at-risk patients, as there is a potential for serious complications including the development of myxedema, as presented in this case study. Traditional treatment in patients who develop myxedema includes the use of steroids and TH replacement therapy. In patients with significant risk factors for coronary disease, caution should be exercised as giving high-dose TH replacement will exert an increase in metabolic demand in an otherwise ischemic prone heart. The use of invasive therapies, such as the use of mechanical circulatory support (MCS), can be considered especially in patients with concomitant myxedema coma and cardiogenic shock as they can help support myocardial function by unloading the left ventricle and reduce diastolic volume. Multiple trials have demonstrated Impella's hemodynamic superiority when compared to IABP in shock and postarrest patients; its use in patients with concomitant myxedema has not been studied [18, 19].

Patients who develop myxedema coma and subsequent cardiovascular collapse are rarely encountered in clinical practice. Myxedema carries a high mortality alone, and concomitant coronary ischemia increases the complexity of the management for these patients. Timing and magnitude of hemodynamic support are imperative to consider given the high mortality and complex nature of these patients. Our case demonstrates the potential for use of MCS for such patients with carefully designed protocols to preemptively detect and ameliorate serious complications, such as bleeding and limb ischemia.

## Abbreviations

CV: Cardiovascular
CT: Computed tomography
CAD: Coronary artery disease
EKG: Electrocardiogram
IABP: Intra-aortic balloon pump
ICM: Iodinated contrast media
LV: Left ventricle
MCS: Mechanical circulatory support
NIS: Sodium/iodide symporter
TH: Thyroid hormone
T4: Thyroxine
TSH: Thyroid-stimulating hormone.
